# Evidence for the role of connexin 43-mediated intercellular communication in the process of intracortical bone resorption via osteocytic osteolysis

**DOI:** 10.1186/1471-2474-15-122

**Published:** 2014-04-09

**Authors:** Shane A Lloyd, Alayna E Loiselle, Yue Zhang, Henry J Donahue

**Affiliations:** 1 Division of Musculoskeletal Sciences, Department of Orthopaedics and Rehabilitation, Penn State College of Medicine, 500 University Drive, Hershey, PA 17033, USA

**Keywords:** Connexin 43, Osteocytic osteolysis, Hindlimb suspension, Unloading, Cortical bone, Porosity

## Abstract

**Background:**

Connexin 43 (Cx43) is the predominant gap junction protein in bone. Mice with a bone-specific deletion of Cx43 (cKO) have an osteopenic cortical phenotype. In a recent study, we demonstrated that cKO mice are resistant to bone loss induced by hindlimb suspension (HLS), an animal model of skeletal unloading. This protective effect occurred primarily as a result of lower osteoclast-mediated bone resorption. Interestingly, we also documented a significant increase in cortical osteocyte apoptosis and reduced sclerostin production. In the present study, we investigated whether osteocytic osteolysis – bone resorption by osteocytes within lacunae – is induced by HLS and the potential effect of Cx43 deficiency on this process during unloading.

**Methods:**

6-month-old male Cx43 cKO or wild-type (WT) mice were subjected to three weeks of HLS (Suspended) or normal loading conditions (Control) (n = 5/group). Lacunar morphology and tartrate-resistant acid phosphatase (TRACP) staining were assessed on sections of femur cortical bone. Experimental groups were compared via two-way ANOVA.

**Results:**

Empty lacunae were 26% larger in cKO-Control vs. WT-Control (p < 0.05), while there was no difference in the size of empty lacunae between Control and Suspended within WT or cKO (p > 0.05). Similarly, there was a trend (p = 0.06) for 11% larger lacunae containing viable osteocytes for cKO-Control vs. WT-Control, with no apparent effect of loading condition. There was no difference in the proportion of TRACP + cells between WT-Control and cKO-Control (p > 0.05); however, WT-Suspended mice had 246% more TRACP + osteocytes compared WT-Control mice (p < 0.05). There was no difference in TRACP staining between cKO-Control and cKO-Suspended (p > 0.05).

**Conclusions:**

Prior to undergoing apoptosis, osteocytes in cKO mice may be actively resorbing their respective lacunae via the process of osteocytic osteolysis. Interestingly, the proportion of TRACP + osteocytes increased dramatically following unloading of WT mice, an effect that was not observed in cKO mice subjected to HLS. The results of the present study provide initial evidence that osteocytic osteolysis is occurring in cortical bone in response to mechanical unloading. Furthermore, Cx43 deficiency appears to protect against osteocytic osteolysis in a manner similar to the inhibition of unloading-induced osteoclast activation that we have documented previously.

## Background

Intercellular communication via gap junctions is one of the critical mediators of the skeletal response to mechanical force. Gap junctions are protein channels that allow for the direct movement of small molecules between adjacent cells [1] or between the cell and extra-cellular environment [2,3]. Gap junction proteins also interact with scaffold proteins and intercellular signaling molecules, such as β-catenin [3-6]. Connexin 43 (Cx43) is the predominant gap junction protein in bone [7] and has been shown to have an important role in the anabolic response to mechanical loading both *in vitro* and *in vivo*[5,8,9]. However, the role of Cx43 in the catabolic response to mechanical unloading (i.e., disuse, lack of weight bearing) is not as well defined.

Mechanical unloading results in significant deleterious effects on the musculoskeletal system. For example, astronauts on four to six month missions aboard the International Space Station lose bone at a rate of 1-2% per month in load bearing bones [10], with incomplete recovery one year after returning to Earth [11]. Unloading-induced bone loss is characterized by an increase in osteoclast-mediated bone resorption (primarily mediated via receptor activator of nuclear factor kappa-B ligand; RANKL [12]) with a decrease in osteoblast-mediated bone formation (primarily mediated by osteocyte-derived sclerostin, an inhibitor of Wnt/β-catenin signaling [13,14]). Unloading-induced bone loss is also associated with prolonged bed rest [15,16], neurological injury or trauma [17], and reduced physical activity in the elderly [18]. Regardless of the specific cause, unloading-induced bone loss may increase fracture risk and increase individual morbidity and mortality [19,20].

Previous studies from our laboratory have subjected mice with an osteoblast/osteocyte-selective deficiency of Cx43 (cKO) to mechanical unloading using the hindlimb suspension (HLS) model [21,22]. HLS has been well-validated as the standard for ground-based simulation of the effects of microgravity and bed rest, including a lack of weight bearing in the hind quarters, cephalic fluid shift, and maintenance of passive muscle activity [23]. Following three weeks of HLS, we were able to detect significant attenuation of trabecular bone loss in cKO mice [21]. In addition, bone formation rate at the femur midshaft of unloaded cKO mice was maintained at baseline levels, as opposed to the characteristic suppression of bone formation observed in unloaded wild-type (WT) mice. In a subsequent study, we found that Cx43 cKO mice subjected to HLS experienced attenuated endocortical osteoclast activity [22]. Similarly, Grimston and colleagues found attenuated bone resorption in Cx43 deficient mice subjected to hindlimb immobilization via botulinum toxin [24].

In addition to osteoclast-mediated bone resorption, recent studies have suggested an important role for osteocytes in bone turnover and skeletal homeostasis via the process of osteocytic osteolysis – direct resorption of bone by osteocytes within their respective lacunae [25-27]. Osteocytes are well positioned for this function given that they are the most abundant cell type in bone and, as opposed to osteoclasts and osteoblasts, they are exposed to a much larger surface area of bone via the lacunar-canalicular network [28].

The baseline cortical phenotype of Cx43 deficient mice is osteopenic, with cortical thinning and marrow expansion due to increased endocortical osteoclast activity [9,24]. We have also documented significantly increased cortical porosity in cKO mice [21]. While not a definitive report, the present study represents an initial investigation of the role of Cx43 in the process of intracortical resorption via osteocytic osteolysis. We subjected WT and Cx43 cKO mice to three weeks of HLS. We found an increased number of empty and enlarged lacunae throughout the cortical bone of cKO mice at baseline, and increased tartrate-resistant acid phosphatase (TRACP)-positive osteocytes in WT mice following HLS. Taken together, these findings suggest a potential role of osteocytic osteolysis in the cortical osteopenic phenotype of Cx43 deficient mice and in the bone loss associated with mechanical unloading.

## Methods

### Mice

Given that global knockout of the Cx43 gene is embryonic lethal [29], we utilized mice with conditional deletion of the gene encoding Connexin 43 (*Gja1*) specifically in osteoblasts and osteocytes. Our strategy for generation of transgenic mice, confirmation of conditional knockout, and determination of the specificity of the Cx43 deletion was described previously [9,21,22]. Briefly, mice expressing Cre recombinase under the control of the human osteocalcin promoter (OC-Cre; Cx43^+/+^) [30] were bred with mice in which *Gja1* is flanked by two loxP sites (Cx43^flx/flx^) [31] to generate OC-Cre; Cx43^flx/+^ mice. We then crossed OC-Cre; Cx43^flx/+^ mice with Cx43^flx/flx^ mice to generate OC-Cre; Cx43^flx/flx^ mice. We then back bred OC-Cre; Cx43^flx/flx^ mice with Cx43^flx/flx^ mice to generate an equal number of OC-Cre; Cx43^flx/flx^ (conditional Cx43 deficient equivalent; cKO) and Cx43^flx/flx^ (wild-type; WT). Mice derived from this breeding strategy were bred with C57BL/6 mice for three generations. Genotyping was performed using DNA isolated from mouse earpieces and appropriate primers [9]. The mice used to generate the data presented in this manuscript were the same mice used in a previous study [22].

### Animal procedures

We utilized six-month-old male Cx43 cKO and WT mice. Mice of this age and sex were selected due to their skeletal maturity and their successful use in our previous studies of mechanical loading [9] and unloading [21,22]. Mice were housed in the central animal facility at the Penn State College of Medicine (Hershey, PA, USA). Mice were fed standard 2018 Teklad Global 18% Protein Rodent Diet (Harlan Laboratories, Inc.; Indianapolis, IN, USA), maintained at a constant temperature of 25°C, and kept on a 12 hour light/dark cycle during all experimental procedures. Mice were housed in standard vivarium enclosures until one-week prior to experimentation, when they were moved to the hindlimb suspension (HLS) enclosures (2 mice per cage) in order to acclimatize under normal loading conditions. WT and cKO mice were then placed into normally loaded (i.e., Control) and HLS (i.e., Suspended) groups (n = 5/group).

The HLS method utilized was a modified version of that first described by Morey-Holton and colleagues [32] and utilized previously by our laboratory [21,22]. The HLS enclosures consisted of a standard rat enclosure with several modifications. The bottom of the cage contained a wire mesh insert with standard bedding placed below. Two metal crossbars were located at either end of the cage, along with water bottles. Under isoflurane anesthesia (2%), two thin strips of bandage tape were then braided around the tail and secured with additional tape at the base of the tail and end. The loose ends of the tape were then fixed to a swivel hook attached to a string. The string was wound around the cross bar at the top of the cage. In this manner, the crossbar could be rotated, thus raising or lowering the hindquarters of the animal to achieve a 30° elevation. This angle of suspension has been previously demonstrated to keep the forelimbs normally-loaded, while minimizing tail tension and animal stress [33]. Two mice were suspended per cage in this manner, although their placement at opposite ends prevented physical contact. Based on our previous experiments, the experimental period of unloading was three weeks [21]. Control mice were housed in this same cage environment, albeit without attachment of the HLS apparatus. The health and activity of the animals was assessed daily by laboratory and veterinary staff. All animal procedures were approved by the Institutional Animal Care and Use Committee at the Penn State College of Medicine (Protocol 2010–117).

### Histology and TRACP staining

Left femurs were harvested immediately after sacrifice and cleaned of all non-osseous tissue. Femurs were then fixed in 10% neutral buffered formalin for 3 days. All femurs were subjected to 7 days of decalcification in sterile 14% ethylenediaminetetraacetic acid at pH 7.4. Femurs were then processed and embedded in paraffin. 5 μm sections (n = 5/group) were used for all analyses. The femur was sectioned longitudinally. We analyzed a 2400 μm lateral section of the cortical bone centered about the midshaft of the femur. We analyzed the entire thickness of cortical bone, from the periosteal to endocortical surface.

Sections were stained with Alcian blue/hematoxylin/Orange G/Eosin (i.e., H&E) or used for TRACP staining. For TRACP staining, following de-waxing and rehydration, slides were incubated in 0.2 M Acetate Buffer (0.2 M Sodium Acetate, 50 mM L(+) tartaric acid; pH 5.0) (Sigma Aldrich; St. Louis, MO, USA) for 20 minutes at room temperature. 0.5 mg/mL Napthol AS-MX phosphate (Sigma) and 1.1 mg/mL Fast Red TR salt (Sigma) were then added to the 0.2 M Acetate Buffer and sections were incubated at 37°C for 2 hours. Sections were then rinsed in distilled water, counterstained in 0.5% Methyl Green (in 100 mM Acetate Buffer; pH 4.2) for 1 minute, washed in running tap water for 5 minutes, dipped in 50% ethanol (Sigma) five times, air dried, and mounted with aqueous Fluoromount-G (Southern Biotech; Birmingham, AL, USA).

All images were acquired using a Nikon Optiphot-2 microscope (Nikon Instruments Inc.; Melville, NY, USA) with 20× objective. Approximately 50–75 images were used to capture the femur diaphysis. These smaller images were stitched together in to one large panorama using Microsoft Image Composite Editor (Microsoft Corporation; Redmond, WA, USA).

H&E-stained sections were used to identify viable osteocytes or empty lacunae. All lacunae in this region were manually traced in order to determine the length of their perimeter (mm) or circularity. Circularity is an index of roundness and is calculated as 4π[(Area)/(Perimeter^2^)]. It can range from 0 (an infinitely elongated polygon) to 1 (a perfect circle). In TRACP-stained sections, the number of TRACP + osteocytes was counted in a 2400 μm length (longitudinal) of cortical bone at the femur midshaft. Measurements were made on n = 5/group. BioQuant Osteo software (v12.5.60, BIOQUANT Image Analysis Corporation; Nashville, TN, USA) was used for all image analysis.

### Immunohistochemistry

Paraffin sections were de-waxed, dehydrated, and underwent antigen retrieval using sodium citrate buffer (pH 6.0). Sections were probed with anti-Cx43 (#SAB4300504, Sigma) antibody diluted 1:500 in normal goat serum, followed by goat anti-rabbit secondary antibody (#PK6101, Vector Laboratories; Burlingame, CA), and staining was visualized with DAB chromogen (Invitrogen). Sections were counterstained with methyl green. 20× images of cortical bone at the femur midshaft were taken.

### Statistical analyses

Statistical analysis was conducted using GraphPad Prism (v5.0f, GraphPad Software Inc.; La Jolla, CA, USA). Data are expressed as mean ± standard error. Statistical evaluation of the data was performed using a two-way ANOVA with post-hoc Student-Newman-Keuls test when the interaction was significant (p < 0.05).

## Results

### Body weight

Consistent with our previous studies [9,21], baseline body weight of mice was not different between WT (31.3±0.8 g) and cKO (31.2±0.9 g) (p > 0.05). Body weight of control mice did not change from day 0 at any time point during the study. Mice subjected to HLS lost a similar amount of body weight, ending the experiment at a similar -5% and -3% of day 0 for WT-Suspended and cKO-Suspended, respectively (p < 0.05). The degree of weight loss during HLS is similar to our previous studies [21,22] and not unexpected following HLS of mature rodents [32].

### Confirmation of connexin 43 deficient phenotype

Using appropriate primers [9], we identified mice as either cKO via positive expression of osteocalcin-driven cre recombinase (OC-Cre) or WT equivalent via negative expression of OC-Cre. Expression of “floxed” *Gja1* was used as a positive control.

**Figure 1:**
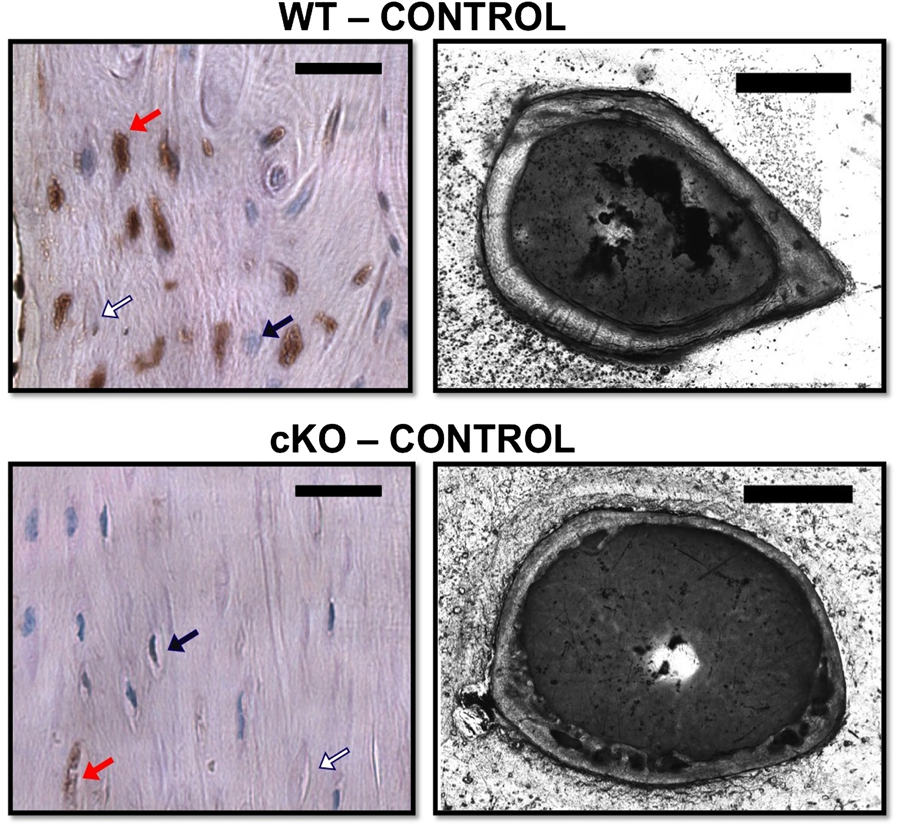
**Cx43 expressing osteocytes.** Left: Representative images demonstrate the relative absence of Cx43+ osteocytes in the femur midshaft cortical bone of cKO mice compared to WT. Red arrow: Cx43+ osteocyte, blue arrow: Cx43- osteocyte, white arrow: empty lacunae. The black bar represents 25 μm. Right: Cross-sections of the femur midshaft taken via light microscope reveal the osteopenic cortical phenotype of cKO mice. The black bar represents 1000 μm.

As expected, there were relatively few Cx43+ osteocytes in the cortical bone of cKO mice compared to WT (Figure 1; left panels). Numerous empty lacunae were noted throughout the cortical bone of cKO mice. These subjective findings are consistent with our previous study, which documented 63% empty lacunae in cKO mice, compared to 9% empty lacunae in WT [22]. A light micrograph cross-section of cortical bone at the femur midshaft highlights the distinctive osteopenic phenotype of cKO mice (Figure 1; right panels), with expanded marrow area and cortical thinning. Specific quantification of the cortical phenotype of Cx43 deficiency has been documented previously by our group [9,21] and others [24,34] using high-resolution microcomputed tomography.

### Cortical lacunae are larger in connexin 43 deficient mice

We found no significant difference between any of the groups with respect to absolute number of lacunae or lacunar density (p > 0.05; Table 1).

**Table 1:** Quantification of lacunar number and density in cortical bone

**Treatment group**	**Total number of lacunae**	**Total sampling area (mm**^**2**^**)**	**Lacunar density (mm**^**-2**^**)**
WT-Control	261 ± 13	0.27 ± 0.01	980 ± 45
WT-Suspended	226 ± 13	0.22 ± 0.02	1054 ± 76
cKO-Control	240 ± 24	0.27 ± 0.04	937 ± 91
cKO-Suspended	253 ± 33	0.23 ± 0.02	1105 ± 64

Lacunae were quantified within a 2400 μm linear region of the mid-diaphysis of the femur. There were no significant differences (p > 0.05) between any of the groups. Comparisons via two-way ANOVA. Mean ± SE.

**Figure 2:**
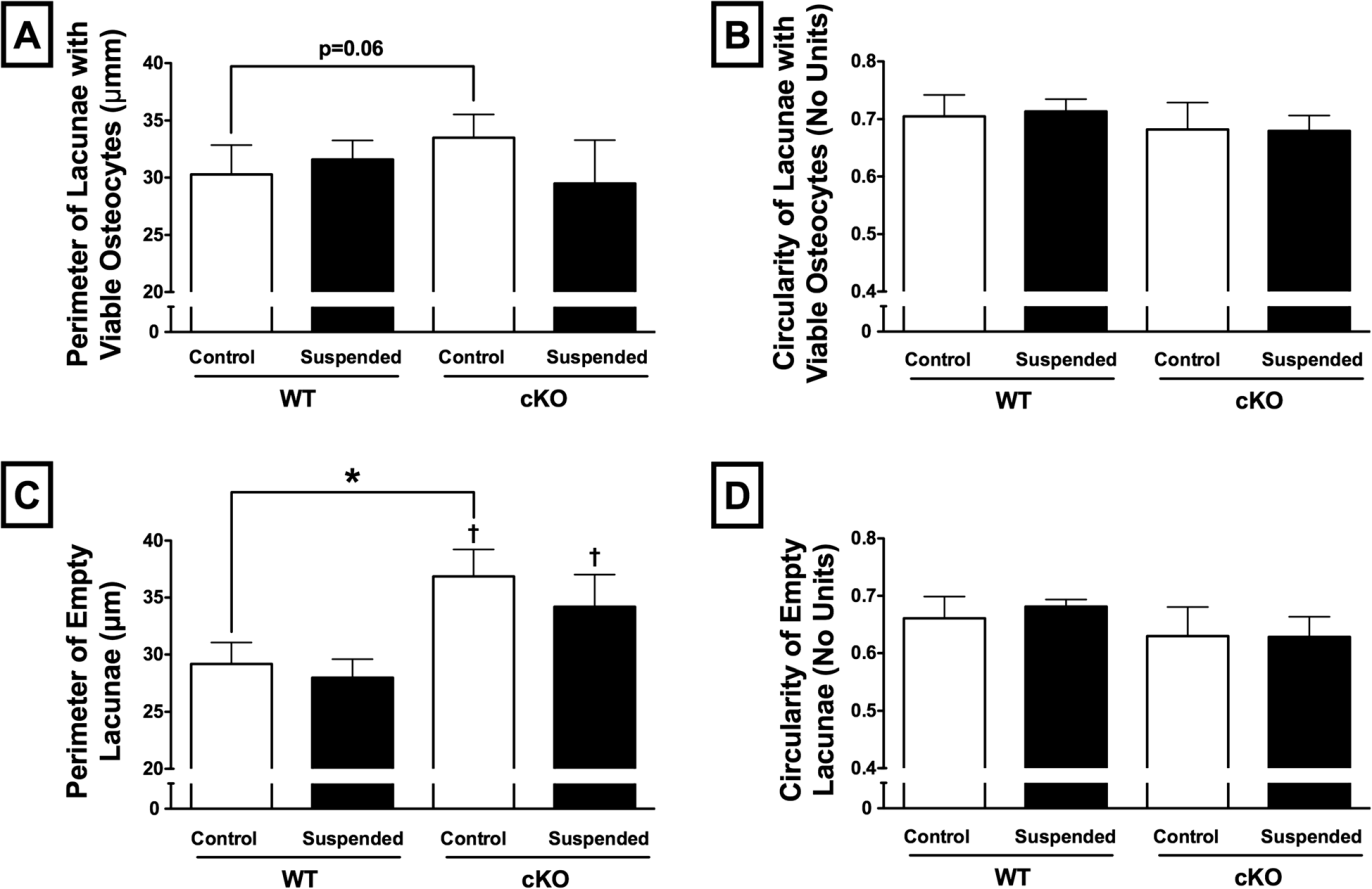
**Osteocyte size and circularity.** Quantification of the relative size and circularity of **(A, B)** lacunae containing viable osteocytes and **(C, D)** empty lacunae in cortical bone from wild-type (WT) and Cx43 conditional knockout (cKO) mice following three weeks of normal loading conditions (Control) or mechanical unloading via hindlimb suspension (Suspended). Lacunae were quantified within a 2400 μm linear region of the mid-diaphysis of the femur. Mean ± SE. * indicates a significant difference (p < 0.05) between Control and Suspended within a genotype, while † indicates a significant difference (p < 0.05) between WT and cKO within a loading condition. n = 5/group.

There was a non-significant trend (p = 0.06) indicating that the size of lacunae containing viable osteocytes was greater in cKO-Control mice relative to WT-Control (+11%) (Figure 2A). As osteocytes resorb the surrounding cortical bone, the shape of their respective lacunae might be expected to become irregular or elongated [35]. However, we were not able to detect a decrease in the relative circularity of lacunae containing viable osteocytes (Figure 2B). Empty lacunae in cKO-Control mice were 26% larger than those in WT-Control (p < 0.05; Figure 2C), although there were no differences between Suspended and Control within a genotype (p > 0.05). There was no difference in the circularity of empty lacunae (Figure 2D).

### TRACP + osteocytes are increased in number during mechanical unloading

**Figure 3:**
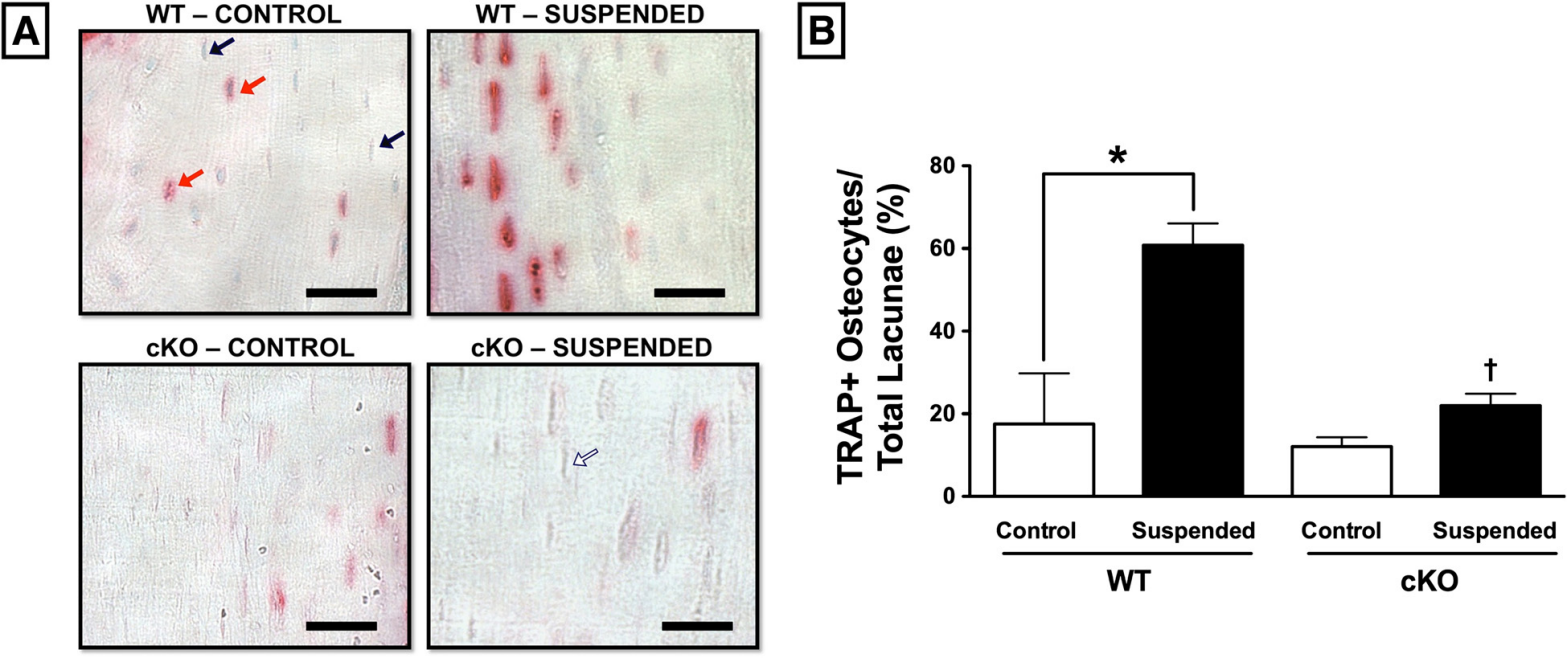
**TRAP expressing osteocytes. (A)** A representative TRACP-stained section of the femur midshaft cortical bone demonstrates the greater number of TRACP + osteocytes in WT-Suspended mice compared to Control, with no difference seen in cKO-Suspended. **(B)** TRACP + osteocytes were quantified relative to total number of lacunae. The black bar represents 25 μm. Red arrow: TRACP + osteocyte, blue arrow: TRACP- osteocyte, white arrow: empty lacunae. Comparisons via two-way ANOVA. Mean ± SE. * indicates a significant difference (p < 0.05) between Control and Suspended within a genotype, while † indicates a significant difference (p < 0.05) between WT and cKO within a loading condition. n = 5/group.

TRACP + osteocytes are an indicator of osteocytic osteolysis [25,36]. TRACP-stained sections of femur cortical bone were used to quantify the number of TRACP + osteocytes, with representative images shown in Figure 3A. There was no difference in the percentage of TRACP + osteocytes quantified relative to total lacunae between WT- and cKO-Control (p > 0.05; Figure 3B). However, we did observe there to be 246% more TRACP + osteocytes in WT-Suspended mice relative to WT-Control (p < 0.05). There was no difference in the proportion of TRACP + osteocytes between cKO-Control and cKO-Suspended (p > 0.05).

## Discussion

Evidence for the process of osteocytic osteolysis comes from studies demonstrating that osteocytes can stain positive for TRACP, an enzyme typically associated with osteoclasts that is critical to their bone resorptive activity [36]. Increased TRACP gene expression has also been demonstrated in osteocyte-enriched bone fractions [25]. In the present study, we found that unloaded WT mice had nearly 2.5 times more TRACP + osteocytes than normally loaded WT mice at the femur midshaft. These findings indicate a potential increase in osteocyte resorptive activity during unloading. Similarly, Blaber and colleagues demonstrated that mice flown for fifteen days on the space shuttle had a greater proportion of TRACP + osteocytes when compared to ground controls [35]. Conversely, Qing colleagues found no increase in TRACP + osteocytes in female CD1 subjected to HLS [25]. It is important to note, however, that the strain (CD1 vs. black 6 background), sex (female vs. male), age (3 months vs. 4 months), duration of unloading (4 weeks vs. 3 weeks), and ground control (attached tail device vs. free ambulation) make a direct comparison to the present study difficult. Regardless, it is clear that additional work is required to reconcile findings in both ground-based controls and in comparison to spaceflight unloading.

Despite an increase in TRACP staining, we were not able to resolve any difference in the relative size or shape of the lacunae in unloaded mice. Qing and colleagues also found no difference between normal control and HLS mice with respect to the size of cortical lacunae [25]. However, it is important to note that HLS is not able to recapitulate all of the effects of true skeletal unloading, a state more closely approximated in microgravity. HLS may result in a relatively lower magnitude of lacunar remodeling that is harder to detect. Indeed, spaceflight studies involving both rats [37] and mice [35,38] have reported the appearance of lacunar enlargement, including increased area, perimeter, and reduced circularity, as well as a significant increase in canalicular diameter. Interestingly, lactating mice have also been shown to have increased lacunar size [25,26].

As opposed to WT mice, there was no increase in TRACP + osteocytes in cKO mice subjected to unloading. Previous studies from our laboratory [22] and another [24] have documented lower cortical osteoclast activity in unloaded cKO mice compared to unloaded WT. Cx43 deficiency appears to effectively desensitize cortical bone to the unloaded state. The results of the present study suggest that Cx43 deficiency has a similar effect on the threshold for initiation of osteocyte resorptive activity during unloading. Osteocytes are the primary source of sclerostin and receptor activator of nuclear factor kappa-β ligand (RANKL) during unloading, resulting in suppression of bone formation and activation of bone resorption, respectively. Lower osteocyte viability induced by Cx43 deficiency [22,34] may partially explain the protective effects of Cx43 deficiency during mechanical unloading. However, prior to undergoing apoptosis, osteocytes may be actively resorbing their respective lacunae, thus contributing to the baseline osteopenic phenotype of Cx43 deficiency. We documented a trend towards increased size of lacunae containing viable osteocytes in cKO-Control mice compared to WT-Control. Furthermore, the size of empty lacunae, which presumably contained an actively resorbing osteocyte for a longer period of time prior to cell death, were a significant 26% larger. The accumulation of larger, empty lacunae may account for the 156% greater cortical porosity we documented in cKO mice versus WT [21].

We were not able to detect a difference in the proportion of TRACP + osteocytes between cKO- and WT-Control; however, it is important to note that TRACP + osteocytes were quantified relative to the total number of lacunae. Given the large proportion of empty lacunae in cKO-Control mice, it is likely that the proportion of TRACP + cells relative to viable cells was actually increased for cKO-Control vs. WT-Control. In addition, it is likely that any TRACP + osteocytes that were actively resorbing and enlarging their lacunae have since died, leaving unstained, empty lacunae behind. Unfortunately, we were not able to quantify TRACP + cells relative to viable osteocytes, as we could not accurately differentiate TRACP- osteocytes from empty lacunae in our histological sections. An important limitation of the present study involves our ability to accurately identify the cells that are occupying the cortical lacunae. A previous study from our laboratory, utilizing tissue sections from these same animals, identified the cells as staining positive for sclerostin [22]. Combined with their location embedded within the bone matrix, it is likely that the cells in question are indeed osteocytes. However, future studies should investigate staining for other osteocyte markers, including sclerostin (Sost) and dentin matrix acidic phosphoprotein 1 (Dmp-1), to confirm absolutely their identity. It would also be interesting to investigate the formation of actin rings in TRACP-positive osteocytes, as this is an essential component of the formation of a resorptive pit by osteoclasts [39].

## Conclusions

The results of the present study provide evidence that osteocytic osteolysis is occurring in cortical lacunae during mechanical unloading. Furthermore, our findings suggest that Cx43 deficiency may protect against osteocytic osteolysis during unloading in a manner similar to the lower endocortical osteoclast activity our group [22] and others [24] have documented previously in unloaded Cx43 deficient mice. It may also be that unloading is not able to further increase osteocytic osteolysis beyond the level already elevated in Cx43 deficient mice at baseline. Important limitations to the present study, including our ability to resolve differences in lacunar size and identify TRACP- osteocytes, would justify additional comprehensive studies of this process. In particular, investigations utilizing high-resolution nanocomputed tomography or electron microscopy are warranted to more accurately quantify lacunar morphology in response to Cx43 deficiency and unloading. Cx43 may represent an important target for the prevention of bone loss associated with aging and disuse.

## Competing interests

The authors declare that they have no competing interests.

## Authors’ contributions

Study design: SAL, HJD; Study conduct: SAL; Data collection: SAL, AEL, YZ; Data interpretation: SAL, AEL, YZ, HJD; Drafting manuscript: SAL, AEL, YZ, HJD; Revising manuscript content: SAL, AEL, YZ, HJD; Approving final version of manuscript: SAL, AEL, YZ, HJD. SAL and HJD take responsibility for the integrity of the data analysis.

## Pre-publication history

The pre-publication history for this paper can be accessed here:

http://www.biomedcentral.com/1471-2474/15/122/prepub
